# Impact of singlehood during pregnancy on dietary intake and birth outcomes- a study in the Norwegian Mother and Child Cohort Study

**DOI:** 10.1186/s12884-014-0396-9

**Published:** 2014-12-05

**Authors:** Jorunn Farbu, Margaretha Haugen, Helle Margrete Meltzer, Anne Lise Brantsæter

**Affiliations:** Division of Environmental Medicine, Norwegian Institute of Public Health, P.O. Box 4404, Nydalen, NO-0403 Oslo Norway

**Keywords:** Marital status, Singlehood, The Norwegian Mother and Child Cohort Study, MoBa, Food frequency questionnaire

## Abstract

**Background:**

Little attention has been given to the impact of singlehood during pregnancy. The aim of this study was to examine the impact of marital status on diet during pregnancy and pregnancy outcome.

**Methods:**

The study population comprised 62,773 women participating in the Norwegian Mother and Child Cohort Study. Marital status was categorised into singles living alone, singles living with parents and married/cohabiting (reference group). Participants answered a general health questionnaire in gestational week 15–17 and a food frequency questionnaire in gestational week 22. We used nonparametric tests to compare dietary intakes by marital status, and multiple logistic regression to estimate odds ratios (OR) and 95% confidence intervals (CI) for infants being small for gestational age (SGA), large for gestational age (LGA), and preterm delivery (defined as delivery before gestational week 37).

**Results:**

Single women living with parents had lower intakes of fruits and vegetables, higher intake of total energy, higher proportion of energy from added sugar, and lower intake of fibre than the reference group. Singles living alone also had a higher intake of added sugar. In both of the single groups, daily smoking was more prevalent than in women living with a partner. In analyses adjusted for maternal age, pre-pregnancy BMI, energy intake, energy contributed by protein, education, income, parity and nausea, single women living alone had increased risk of SGA with OR = 1.27 (95% CI: 1.05, 1.55). When smoking was included among the confounding variables, the association was no longer significant. Likewise, singles living alone had increased risk of preterm delivery, with OR = 1.32 (95% CI: 1.01, 1.72) in a partly adjusted model, but the association did not remain significant in a model fully adjusted for confounding variables.

**Conclusions:**

Single mothers had lower dietary quality and included more smokers than women who lived with a partner. Single mothers living alone had higher prevalence of SGA and preterm delivery, but the associations with adverse pregnancy outcomes were confounded by other variables. This study shows that single mothers should be given special attention during antenatal care and counselling.

## Background

There have been major changes in household composition the last decades, with increased proportion of children being born to single mothers. According to Statistics Norway, 13% of all children were born to single mothers in 2013, as compared to 9% in 1994 [1]. Marital status has been associated with adverse health behaviour, including poorer eating habits, with higher prevalence of cardio-vascular disease, type II diabetes, obesity and mental illness in single households than in families [2-4]. A systematic review and meta-analysis of twenty-one studies, published in 2011 concluded that single women had increased risk of adverse pregnancy outcomes, including preterm delivery, low birth weight and small for gestational age infants [5].

Foetal development is characterized by rapid growth, sensitive to quality and quantity of nutrients consumed during pregnancy [6] and maternal diet may impact the long-term health of both mothers and children [7-10]. Birth weight is a marker of foetal growth and a predictor of infant survival and health status. Birth weight depends on gestational length and the outcomes ‘small for gestational age’ (SGA) and ‘large for gestational age (LGA)’ are used to identify high risk infants. Maternal intake of micronutrients [11,12], macronutrients [13], as well as food intakes [14-17] has been associated with pregnancy outcomes including SGA, LGA and gestational length.

Studies have shown that pregnant women often fail to meet their respective countries’ dietary recommendations [18-20], but few have reported dietary quality or food intake in pregnant single women [21,22]. To the best of our knowledge, no previous studies have examined the associations between marital status and pregnancy outcomes taking maternal diet into account. The objective of the present study was therefore to examine the impact of marital status on diet during pregnancy and the pregnancy outcomes SGA, LGA and preterm delivery.

## Methods

### Population and study design

The Norwegian Mother and Child Cohort Study (MoBa) is a prospective population-based pregnancy cohort study conducted by the Norwegian Institute of Public Health. Participants were recruited from all over Norway from 1999–2008. The women consented to participation in 40.6% of the pregnancies. The cohort now includes 114,500 children, 95,200 mothers and 75,200 fathers [23]. The study aims to follow the children up to 14 years of age through questionnaires, and later in life through Norway’s many health registries. Women were recruited to the study through a postal invitation in connection with their first routine ultrasound control at week 17–18 of pregnancy. Data were collected through comprehensive questionnaires and blood and urine samples to provide researchers with a wide range of data for future hypothesis testing. Nearly all participants were of Caucasian ethnicity. The data from MoBa were linked to the Medical Birth Registry of Norway (MBRN), in which all births and stillbirths have been registered since 1967 [24]. Informed consent was contained from all participants before study entry. The study was approved by the Regional Committee for Ethics in Medical Research and the Data Inspectorate in Norway.

The current study uses the quality-assured data files released for research in 2009 (version 4). Data collected for this study were collected from questionnaire 1 (Q1) and questionnaire 2 (Q2). Q1, received in pregnancy weeks 13–15, comprised socio-demographic information and general health, while Q2 is a semi quantitative food frequency questionnaire sent to the participants around week 17–22 of pregnancy.

**Figure 1:**
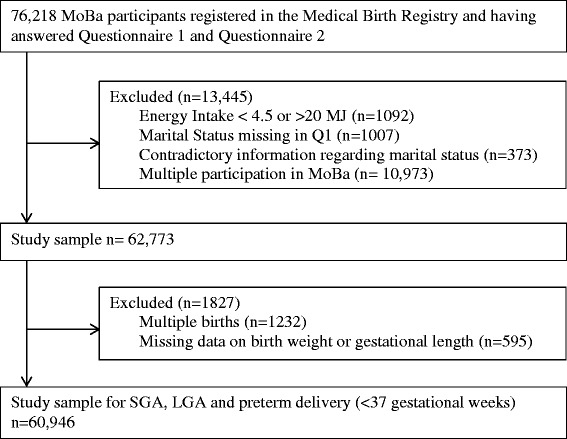
**Flow diagram for inclusion of participants.**

The participants in the present study were recruited between 2002 and 2007. In total, 62,773 women were eligible to participate in the current study. The women included were those who participated for the first time and had answered both Q1 and Q2. Other inclusion criteria were: having reported a valid energy intake [25] and having reported the same marital status in the MBRN register at the time of delivery as in the first MoBa questionnaire. A flow diagram for inclusion of participants is presented in Figure 1. For studying the association between marital status and the birth outcomes SGA, LGA and preterm delivery, we excluded women with multiple pregnancies (twins/triplets, n = 1232) and those with missing data on infant birth weight or gestational length (n = 595), resulting in 60,946 women. Women with missing information (n = 1007) or contradictory information (n = 373) on marital status (Figure 1) were categorized as a “missing marital information” group and included in a sensitivity analysis.

### Definition of marital status

The participants were divided into singles living alone (SA), singles living with parents (SP) and married/cohabiting (M/C). The single category was divided in two due to the differences in age and living conditions between these two sub-groups.

### Dietary information

The MoBa FFQ (downloadable from www.fhi.no/dokumenter/011fbd699d.pdf) is a semi-quantitative questionnaire that asked about the intake of 255 food items and was specifically designed to capture dietary habits and intake of dietary supplements during the first 4–5 months of pregnancy [25]. The questionnaires were optically read. Frequencies were converted into food intakes and nutrient calculations were performed with the use of FoodCalc [26] and the Norwegian food composition table. A validation study showed that compared to a dietary reference method and biological markers of intake, the FFQ produces a realistic estimate of the habitual intake and is a valid tool for ranking pregnant women according to high and low intakes of energy, nutrients and food [27-29].

### Pregnancy outcomes

The pregnancy outcomes included in the present study were a) small for gestational age (SGA), b) large for gestational age (LGA) and c) preterm delivery. The variables SGA and LGA were calculated from the 10th percentile and 90th percentile of birth weight within gestational week for nulliparous and multiparous pregnancies respectively. Preterm delivery was defined as pregnancies with gestational length shorter than 37 complete weeks. The information related to gestational length and infant birth weight was retrieved from the MBRN. Gestational length was calculated from ultrasound measurements at week 17–18, with the exception of a few women with missing ultrasound information. For these women, gestational length was calculated from the date of their last menstruation.

### Other variables

Maternal age at delivery reported in MBRN was used as a continuous variable with exception of descriptive statistics, for which it was divided into five categories (<20, 20–24, 25–29, 30–34, and ≥35). BMI was calculated from self-reported height and weight before the pregnancy reported in Q1 and categorized according to the World Health Organization classification as normal (18.5-24.9 kg/m^2^), underweight (<18.5 kg/m^2^), overweight (25.0-29.9 kg/m^2^) and obese (≥30.0 kg/m^2^). Education was divided into four categories (<12 years, 12 years, 13–16 years and ≥17 years). Smoking habits during the first part of pregnancy were reported in Q1. We categorised smoking into three groups: daily smokers, occasional smokers and non-smokers. Q1 included a short version of the Hopkins Symptom Checklist. We included a dichotomous variable denoting whether women had experienced feeling depressed or sad for a continuous period of more than two weeks during the first part of pregnancy [30]. The variable was used as an indicator of mental wellbeing.

### Statistical methods

Initially data were analysed for missing values and normality of continuous data. For the maternal demographics, chi-square was used for nominal data. Food and nutrient intakes are presented as medians, 5 and 95 percentiles (P5, P95). For all continuous variables, the Kruskal-Wallis test was chosen when comparing three groups, and Mann–Whitney-U test was chosen when comparing two groups due to the differences in the group sizes. Logistic regression was used to estimate odds ratios (OR) and 95% confidence intervals (CI). Each of the pregnancy outcomes SGA, LGA and preterm delivery were modelled as dependent variables and adjusted for dietary variables (total energy and nutrient intakes) and potential confounding variables (maternal characteristics presented in Table 1). Dietary intakes and confounding variables were included in the models if they were associated both with marital status and the outcome with p < 0.100. The following variables were included in the final models: total energy intake, energy contributed by protein, maternal pre-pregnancy BMI, education, income, parity, age at delivery, nausea at the time of filling in the FFQ and smoking during pregnancy. All analyses were performed using SPSS version 17. All p-values were two sided and values <0.05 were considered significant.

**Table 1:** **Maternal characteristics by marital status (n = 62,773)**

	**Married/cohabiting (n = 61,646)**		**Single living alone (n = 909)**		**Single living with parents (n = 218)**		**p-value**
	**N or Mean**	**% or SD**	**N or Mean**	**% or SD**	**N or Mean**	**% or SD**	
Age, years	30.1	4.5	29.5	6.4	21.6	4.6	< 0.001^*^
Age in categories							< 0.001^†^
< 20	391	0.6	36	4.0	83	38.1	
20-24	6295	10.2	214	23.5	92	42.2	
25-29	21,184	34.4	210	23.1	25	11.5	
30-34	26,430	42.9	266	29.3	15	6.9	
≥35	7346	11.9	183	20.1	3	1.4	
BMI prior to pregnancy, kg/m^2^	24.1	4.3	24.3	5.1	23.8	4.9	0.047^‡^
BMI in categories							< 0.001^†^
<18.5	1730	2.8	48	5.3	16	7.3	
18.5- 24.9	39,380	63.9	525	57.8	137	62.8	
25 -29.9	13,176	21.4	182	20.0	29	13.3	
30-34.9	4204	6.8	75	8.3	21	9.6	
≥35	1560	2.5	43	4.7	8	3.7	
Missing	1596	2.6	36	4.0	7	3.2	
Education							< 0.001^†^
< 12 years	11,865	19.2	370	40.7	135	61.9	
12 years	7448	12.1	140	15.4	48	22.0	
13-16 years	26,056	42.3	236	26.0	19	8.7	
17 + years	15,019	24.4	144	15.8	8	3.7	
Missing	1258	2.0	19	2.1	8	3.7	
Income NOK							< 0.001^†^
None	1298	2.2	54	6.3	54	27.4	
<150,000	9211	15.5	285	33.1	98	49.7	
150-199,000	6660	11.2	113	13.1	20	10.2	
200-299,000	21,431	36.0	211	24.5	20	10.2	
300-399,000	14,689	24.6	127	14.8	3	1.4	
≥400,000	6320	10.6	71	8.2	2	1.0	
Missing	2037^§^		48 ^§^		21^§^		
Smoking in pregnancy							< 0.001^†^
Daily	3140	5.1	203	22.3	53	24.3	
Occasional	1631	2.6	73	8.0	28	12.8	
Non smokers	56,441	91.6	623	68.5	134	61.5	
Missing	434	0.7	10	1.1	3	1.4	
Parity							< 0.001^†^
Primiparous	32,556	52.8	592	65.1	204	93.6	
Multiparous	29,090	47.2	317	34.9	14	6.4	
Nausea at time of FFQ							< 0.008^†^
Yes	7042	11.4	126	13.9	35	16.1	
Have felt depressed							< 0.001^†^
Yes	30,147	48.9	609	67.0	128	58.7	
Missing	655	1.1	16	1.8	6	2.8	

*SD*, Standard deviation.

^*^One-way Anova with post hoc tests.

^†^χ^2^ test.

^‡^Kruskal-Wallis test.

^§^Not included in the percent distribution.

## Results

Of the 62,773 women in this study, 61,646 (98.2%) were married/cohabiting, 218 (0.3%) were single living with their parents, and 909 (1.5%) were single living alone.

Maternal characteristics differed substantial by marital status (Table 1). The single groups were younger, and had lower education and income than the married/cohabiting group. In particular, the prevalence of smoking was higher in the single groups. Singles also reported higher prevalence of feeling depressed or sad for a prolonged time. There were major differences also between the two single groups, with singles living alone representing a more heterogeneous group than singles living with parents (Table 1).

Food intakes differed substantial by marital status (Table 2). Compared to married/cohabiting women, singles living alone had higher intake of full fat milk and lower intake of meat, while singles living with parents had lower intakes of vegetables and whole grain products, and higher intakes of full fat milk and sugared sweetened drinks. Analyses of selected nutrient intakes by marital status reflected the differences in food intake (Table 3). Both single groups had higher energy intake, particularly energy contributed by added sugar, but also less energy contributed by protein. The singles living alone had higher intakes of saturated fat and both single groups had lower intake of dietary fibre than the married/cohabiting group, whereas the singles living with parents had lower intake of folate both from food and supplements.

**Table 2:** **Food intakes (g/day) by marital status (n = 62,773)**

	**Married/cohabiting (M/C) n = 61,646**			**Singles living alone (SA) n = 909**			SA vs M/C *	**Singles living with parents (SP) n = 218**			SP vs M/C *	SA vs SP*
	**Median**	**P5**	**P95**	**Median**	**P5**	**P95**	**p-value**	**Median**	**P5**	**P95**	**p-value**	**p-value**
Dairy all	420	50	1160	410	50	1330	0.222	460	50	1740	0.012	0.117
Full fat milk	2	0	200	13	0	400	<0.001	31	0	820	<0.001	<0.001
Low fat milk	250	0	880	210	0	1200	0.024	200	0	1200	0.590	0.714
Cheese	17	2	61	15	1	65	0.001	10	1	56	<0.001	<0.001
White bread	97	0	290	86	0	320	0.957	120	1	360	0.001	0.002
Dark bread	45	0	270	45	0	270	0.295	6	0	270	<0.001	<0.001
Cereals, porridge	11	0	110	10	0	115	0.017	7	0	90	<0.001	0.022
Fruit	221	50	620	203	34	680	0.110	186	0	90	0.006	0.091
Vegetables	135	43	340	127	33	370	0.065	100	15	330	<0.001	<0.001
Meat all	99	55	148	93	44	155	<0.001	96	47	160	0.285	0.179
Poultry	17	0	47	15	0	48	<0.001	12	0	46	<0.001	0.030
Seafood all	34	6	76	35	0	83	0.397	30	0	81	0.232	0.184
Fatty fish	8	0	38	8	0	40	0.291	6	0	32	<0.001	0.002
Pizza, taco	18	13	25	18	11	26	0.006	20	13	28	0.047	0.007
Potatoes, boiled or mashed	38	1	100	29	4	120	<0.001	56	10	130	<0.001	<0.001
French fries, fried potatoes	10	0	17	10	0	17	0.719	10	0	17	<0.001	<0.001
Sugar sweetened drinks	55	0	610	67	0	1020	0.081	140	0	1500	<0.001	<0.001
Coffee	4	0	107	5	5	150	0.034	0	0	16	<0.001	<0.001
Cakes	6	0	22	6	0	27	<0.001	6	0	29	0.003	0.587
Sweets	17	1	80	16	0	89	0.028	15	0	105	0.305	0.980
Salty snacks	12	2	36	10	0	45	0.002	13	0	63	0.133	0.014
Olive oil	0.4	0	3.0	0.2	0	2.1	<0.001	0.1	0	2.1	<0.001	0.001

*Mann–Whitney U test, P5, 5^th^ percentile; P95, 95^th^ percentile.

**Table 3:** **Selected nutrient intakes by marital status**

	**Married/cohabiting (M/C) n = 61,646**			**Singles living alone (SA) n = 909**			SA vs M/C*	**Singles living with parents (SP) n = 218**			SP vs M/C*	SA vs SP*
	**Median**	**P5**	**P95**	**Median**	**P5**	**P95**	**p-value**	**Median**	**P5**	**P95**	**p-value**	**p-value**
Energy, MJ	9.4	6.1	14.6	9.7	5.8	15.9	0.002	10.1	5.6	16.6	<0.001	0.081
Protein energy %	15.4	12.1	19.0	15.1	11.3	19.1	<0.001	14.7	10.9	19.3	<0.001	0.002
Fat energy %	30.3	23.2	37.9	30.5	22.9	39.7	0.147	31.0	21.5	37.5	0.987	0.477
Carbohydrate energy %	53.8	46.3	61.8	53.9	44.5	63.1	0.707	54.1	46.8	64.0	0.143	0.145
Added sugar energy %	9.8	4.2	19.7	10.1	4.1	23.7	0.002	12.1	4.4	27.7	<0.001	0.001
Saturated fat g/10 MJ	31.4	23.0	41.2	32.2	22.8	43.0	<0.001	31.7	22.3	43.2	0.182	0.682
Fibre, g/10 MJ	31.3	21.1	47.7	30.1	18.3	45.9	<0.001	27.5	14.1	39.6	<0.001	<0.001
Vitamin D from food, μg/10 MJ	3.3	1.1	6.7	3.3	1.1	7.2	0.687	3.2	0.8	7.8	0.421	0.367
Total Vitamin D^†^, μg/d	7.9	1.8	30.5	8.1	1.5	32.6	0.783	7.1	1.2	3.19	0.039	0.061
Folate food, μg/10 MJ	277	190	412	276	178	423	0.148	263	162	391	<0.001	0.011
Total folate^†^, μg/d	445	174	988	426	151	1010	0.023	338	142	926	<0.001	0.001
n-3 from supplements^‡^, g/day	0.40	0.06	2.40	0.60	0.08	2.94	<0.001	0.41	0.05	2.79	0.869	0.109
Calcium, g/10 MJ	1.05	0.63	1.63	1.04	0.60	1.72	0.847	1.00	0.55	1.92	0.184	0.303
Magnesium, mg/10 MJ	413	326	512	410	303	530	0.124	388	301	495	0.001	<0 · 001

Energy %, percentage of energy contributed by nutrient.

P5, 5^th^ percentile; P95, 95^th^ percentile.

^*^Mann–Whitney U test.

^†^Including supplements.

^‡^Intake in supplements users only.

Analysis of associations between marital status and pregnancy outcome (Table 4) showed that singles living alone had significantly higher risk of SGA and preterm delivery than the married/cohabiting women after adjusting for the nutrition related variables (maternal BMI, total energy intake and energy contributed by protein, Table 4, Model 1). For SGA, the association remained significant after additional adjustment for maternal education, income, parity and age of delivery (OR: 1.27, 95% CI: 1.05, 1.55) (Table 4, Model 2). However, after adjusting also for maternal smoking the association did no longer remain significant (Table 4, Model 3). Likewise, singles living alone had increased risk of preterm delivery in the model adjusted for nutrition related variables, with OR = 1.32 (95% CI: 1.01, 1.72) (Table 4, Model 1), but the association did not remain significant when additional confounding variables were included (Table 4, Models 2 and 3).

**Table 4:** **Associations between marital status and pregnancy outcomes in 60,946 women**

	**Total n**	**n (%)**	**Unadjusted OR (95% CI)**	Model 1* Adjusted OR (95% CI)	**Model 2** ^**†**^ **Adjusted OR (95% CI)**	**Model 3** ^**‡**^ **Adjusted OR (95% CI)**
**Small for gestational age baby**						
Married/cohabiting	59,845	6289 (10.5)	1	1	1	1
Singles living alone	888	123 (13.9)	1.37 (1.13, 1.66)	1.36 (1.12, 1.64)	1.27 (1.05, 1.55)	1.10 (0.90, 1.34)
Singles living with parents	213	22 (10.3)	0.98 (0.63, 1.53)	0.93 (0.59, 1.45)	0.96 (0.61, 1.50)	0.82 (0.52, 1.29)
**Large for gestational age baby**						
Married/cohabiting	59,845	5839 (9.8)	1	1	1	1
Singles living alone	888	80 (9.0)	0.92 (0.73, 1.15)	0.88 (0.70, 1.11)	0.87 (0.69, 1.10)	0.94 (0.75, 1.20)
Singles living with parents	213	24 (11.3)	1.17 (0.77, 1.80)	1.20 (0.78, 1.84)	1.11 (0.72, 1.71)	1.20 (0.78, 1.87)
**Preterm delivery (<37 weeks)**						
Married/cohabiting	59,845	2966 (5.0)	1	1	1	1
Singles living alone	888	59 (6.6)	1.36 (1.05, 1.78)	1.32 (1.01, 1.72)	1.17 (0.89, 1.53)	1.15 (0.88, 1.51)
Singles living with parents	213	12 (5.6)	1.14 (0.64, 2.05)	1.09 (0.61, 1.95)	0.91 (0.50, 1.64)	0.89 (0.49, 1.61)

^*^Model 1: adjusted for maternal pre-pregnancy BMI, total energy intake and energy contributed by protein.

^†^Model 2: additional adjusted for maternal education, income, parity, age at delivery and nausea at the time of filling in the FFQ.

^‡^Model 3: additional adjusted for maternal smoking during pregnancy.

In a sensitivity analysis we included women with missing or inconsistent information on marital status (n 1380) in a ‘missing marital information group’. Comparison of age, education, smoking status, parity and the prevalence of pregnancy outcomes in this group and the three marital groups in the study, showed that the missing group comprised women from all three groups. Compared to the reference group (married/cohabiting), the missing group was not associated with any pregnancy outcomes (data not shown). Furthermore, including the missing group in the analysis of marital status versus pregnancy outcomes did not change the associations reported in Table 4.

## Discussion

The main finding of the present study was the differences in dietary quality with regard to marital status. Singles living with parents and singles living alone had lower nutrient dense diets than women who were married/cohabiting. Singles living alone had higher risk of SGA and preterm delivery than women living with a partner. However, the associations were confounded by other socioeconomic and lifestyle variables, in particular maternal education and smoking.

The observed difference in dietary quality between pregnant single women and those living with a partner (Tables 2 and 3) is in accordance with previous studies linking diet to marital status [21,22]. Northstone et al. examined associations between dietary patterns in pregnancy and socio-demographic and lifestyle factors in a British cohort, and reported lower adherence to a ‘health conscious’ and higher adherence to a ‘processed’ dietary pattern in women who were single than in non-singles [21]. Similar associations were seen for education. Another recent study using data from MoBa examined whether loneliness, marital status, and other factors were associated with consumption of sodas and juices. Their results showed that being married or cohabiting was associated with a lower intake of sugar-containing beverages [22].

The difference between the two single groups observed in our study may partly be explained by age, education and socioeconomic status. Adolescents more often have energy dense and nutrient poor diets [31,32]. In non-pregnant populations it has been shown that children’s eating patterns mirror what is available at home, and that parental education, particularly maternal education, is closely associated with adolescents’ dietary habits [4,33,34].

Marital status has been associated with health, health related behaviours and birth outcomes [2,3,5]. A systematic review and meta-analysis of 21 cohort studies in developed countries concluded that compared to women who were married, single women had increased risk of preterm delivery, low birth weight and SGA [5]. However, there was large heterogeneity among the studies, and only some studies included adjustment for socioeconomic variables.

In the current study, associations between marital status and pregnancy outcomes were clearly modified by confounding variables, particularly smoking and education (Table 4). Similar to our study, a study not included in the review, with 304 unmarried and the same number of matched controls, did not find any associations between marital status and risk of preterm delivery or SGA [35]. It could be questioned whether marital status is merely a marker of socioeconomic status. Although Norway is believed to be an egalitarian society, several studies have shown that socioeconomic variables, particularly maternal education and household income, are associated with health behaviour and pregnancy outcomes [34,36-38].

Singles living alone represented a more diverse group in terms of age, education and economy than single women living with parents. Older and more educated single mothers might have a less stress-related burden in their pregnancies, and in the highest income category (≥400,000 NOK) there were almost as many single women living alone (8.2%) as married/cohabiting (10.6%). The percentage of mothers aged 35 years or more were highest in the singles living alone group. A previous MoBa study reported that women giving birth to their first baby at an advanced or very advanced age compose a heterogeneous group characterized by either socioeconomic prosperity or vulnerability. Single status was among the socio-demographic factors correlated with giving birth at an advanced age [39]. Although single mothers only represented 1.8 % of the total cohort in this study, single mothers constituted 13 % of all pregnant women in 2013 in the general population and is most likely an increasing group [1].

The main strengths of this study include the large sample size representing women from all regions of Norway, the prospective design, and the comprehensive information about the maternal diet and a wide range of potential confounding factors. However, the low participation rate in MoBa is a concern (40.6%), with underrepresentation of women aged less than 25 years, smokers, those living alone, those with more than two previous births and those with previous stillbirths [40]. The potential selection bias in MoBa has been evaluated, and despite differences in prevalence estimates, associations between eight exposures and outcomes did not differ between MoBa and a representative sample from the national birth registry [40].

The MoBa FFQ has been thoroughly validated, but the FFQ method has several limitations. Answering a FFQ challenges the respondents with rather complex cognitive skills, such as reporting the average intake of a given food or dish during the time period covered. FFQ’s are subject to recall bias, and are not a precise instrument to estimate nutrient intakes on an individual level. Nevertheless, FFQs have proved to be an appropriate method to capture an image of the distribution of the intake of energy, nutrients and foods on a population level [27,41]. Although MoBa participants were not representative and have a healthier lifestyle than the general population of pregnant women, few women fulfil the dietary recommendations [20].

Maternal smoking, poor gestational nutrition and low pre-pregnancy weight are the most important modifiable risk factors for foetal growth restriction in developed countries [42]. In our study, all of these factors were more prevalent in the two single groups than in married/cohabiting women (Table 1).

## Conclusions

The current study showed that single mothers had lower dietary quality than women who lived with a partner. This was reflected by higher intake of energy, particularly energy contributed by added sugar, lower intake of dietary fibre and lower intake of energy contributed by protein. Single mothers living alone had higher prevalence of SGA and preterm delivery, but the associations with the adverse pregnancy outcomes were confounded by other variables, particularly smoking and educational attainment. Our results show that the risk is not equally distributed among single women. This study shows that single mothers should be given special attention during antenatal care and counselling.

## Abbreviations

BMI: Body mass index
CI: Confidence interval
FFQ: Food frequency questionnaire
MoBa: The Norwegian Mother and Child Cohort Study
MBRN: Medical birth registry of Norway
SP: Singles living with parents
SA: Singles living alone
M/C: Married/cohabiting
NOK: Norwegian crowns, currency
SGA: Small for gestational age
LGA: Large for gestational age
